# Identification of the trade-off between speed and efficiency in undulatory swimming using a bio-inspired robot

**DOI:** 10.1038/s41598-023-41074-9

**Published:** 2023-09-12

**Authors:** Alexandros Anastasiadis, Laura Paez, Kamilo Melo, Eric D. Tytell, Auke J. Ijspeert, Karen Mulleners

**Affiliations:** 1 Unsteady Flow Diagnostics Laboratory, Institute of Mechanical Engineering, École Polytechnique Fédérale de Lausanne (EPFL), 1015 Lausanne, Switzerland; 2 Biorobotics Laboratory, Institute of Bioengineering, École Polytechnique Fédérale de Lausanne (EPFL), 1015 Lausanne, Switzerland; 3 KM-RoBoTa, 1020 Renens, Switzerland; 4 Department of Biology, Tufts University, Medford, MA 02155 USA

**Keywords:** Mechanical engineering, Fluid dynamics, Biomechanics

## Abstract

Anguilliform swimmers, like eels or lampreys, are highly efficient swimmers. Key to understanding their performances is the relationship between the body’s kinematics and resulting swimming speed and efficiency. But, we cannot prescribe kinematics to living fish, and it is challenging to measure their power consumption. Here, we characterise the swimming speed and cost of transport of a free-swimming undulatory bio-inspired robot as we vary its kinematic parameters, including joint amplitude, body wavelength, and frequency. We identify a trade-off between speed and efficiency. Speed, in terms of stride length, increases for increasing maximum tail angle, described by the newly proposed specific tail amplitude and reaches a maximum value around the specific tail amplitude of unity. Efficiency, in terms of the cost of transport, is affected by the whole-body motion. Cost of transport decreases for increasing travelling wave-like kinematics, and lower specific tail amplitudes. Our results suggest that live eels tend to choose efficiency over speed and provide insights into the key characteristics affecting undulatory swimming performance.

## Introduction

Eels demonstrate exceptional swimming efficiency during their migration period, travelling thousands of kilometres without feeding. Eels typically use 4 to 6 times less energy than trout when swimming at the same speed^1–3^. The high efficiency of these swimmers makes them an interesting study object and a potential inspiration for engineering applications like swimming robots. Robotic platforms have been inspired by natural undulatory swimmers and can be used to inform our understanding of animal swimming. It requires special training and dedication to try to control the swimming kinematics of living animals, and it is challenging to measure their power consumption^4^. Instead, we can use robots to measure different quantities that determine swimming performance, such as swimming speed, energy efficiency (cost of transport), and acceleration. We can examine how these quantities depend on body kinematics, investigate underlying trade-offs, and replicate high swimming speeds and accelerations^5,6^. The visualisation of the wake flow generated by fish-like robots has revealed that the creation of coherent vortex patterns that match the wake patterns behind fish increases swimming performance^7–9^.

Combining animal observations, robotic studies, and numerical simulations has enhanced our understanding of anguilliform swimming. Anguilliform swimmers likely achieve high swimming efficiencies by controlling their body undulation and interaction with the surrounding fluid. Eels and other elongated anguilliform swimmers undulate their bodies with travelling waves that are usually shorter than their body length^10–12^. Compared to other fishes, anguilliform swimmers use whole-body undulations and have a waveform with higher curvature in the anterior two-thirds of their body^12^. At slow swimming speeds, eels have low undulation amplitudes in the anterior part of their body^11^. To increase their swimming speed, eels keep their tail amplitude relatively constant and proportionally increase their tail beat frequency and the undulation amplitude of the anterior part of their body^13^. The anterior body undulation leads to the formation of vortices close to the body that are advected by the body undulation towards the tail, where they shed into the wake and enhance the downstream momentum flux^14,15^. Vortices are low pressure regions, and when they pass on the inside of a strong body curvature, they might also aid the body undulation and push the fish forward^16,17^. A similar process of anterior vortex formation, advection, and shedding is observed for flexible panels with low undulation amplitudes, explaining their high propulsive efficiency^18^. Numerical optimisation of anguilliform swimmers revealed that a significant contribution of the anterior part of the body is indeed crucial for efficient thrust production^19^.

For anguilliform swimmers, proper analysis of the body undulation, including its wavelength, amplitude, and frequency, is essential for understanding and replicating efficient swimming performances. Anguilliform and carangiform swimmers change their tail beat frequency to modulate speed, keeping tail beat amplitude nearly constant^12,20,21^. Most fishes seem to maintain a consistent body wavelength across swimming speeds. Eels, however, are observed to swim with substantially different wavelengths ($\lambda$) at different swimming speeds ^14,22^. The wavelength of American eels ranges from $\lambda ={0.4}\,\hbox {to}\,{0.7}$ body lengths (*L*) at speeds from ${0.5}\,\,\hbox {to}\,{2.0}\,{\textrm{L}}/{\textrm{s}}$^22^ and the wavelength of European eels ranges from $\lambda ={0.69}\,\,\hbox {to}\,{0.87}\,\textrm{L}$ at speeds from ${1}\,\,\hbox {to}\,{1.5}\,\textrm{L}/{\textrm{s}}$^14^. Fish also adjust their wavelength when encountering a von-Karman vortex street^23^. Numerical simulations of waving foils suggest that an increase in wavelength increases the mean thrust produced, and a decrease in the wavelength leads to smaller fluctuations in the thrust^24–26^. An optimum wavelength-to-tail amplitude ratio results in maximum thrust production in a robotic platform and numerical simulations^27,28^, and most undulatory natural swimmers swim near this optimum ratio^29^.

Systematic experiments are desirable to analyse the effect of the undulation characteristics on the performance of anguilliform swimmers, to broaden our understanding of fish swimming, and to guide the design of efficient undulatory swimming robots. In particular, such experiments can provide insights into different quantities that determine swimming performance (e.g. swimming speed, energy efficiency, acceleration) and trade-offs between these quantities that fishes ^30^ and fish-like robots ^31^ need to make when choosing swimming kinematics. Most fish actively control their undulation amplitude and wavelength by muscle action^32,33^. It is tempting to assume that the observed kinematics in fishes are a result of energy efficiency, but other criteria might affect the observed fish motion^4^. The mathematical model by Porez et al.^34^ predicts an undulatory swimmer’s speed based on its input kinematics but does not provide insight into the swimming cost of transport. Novel bio-inspired robots can replicate the swimming behaviour of live animals, but they can also be used to measure the performance of swimming kinematics that are unobserved in animals. Furthermore, we can more easily assess the power consumption of robotic swimmers than that of living animals.

In this study, we focus on two key criteria of swimming performance: swimming speed and cost of transport. We present a comprehensive characterisation of the effect of undulatory swimming kinematics, including the wavelength, amplitude, and frequency of the undulation, on the performance criteria using a bio-inspired anguilliform robotic platform. We present performance maps of speed and cost of transport for a sweep of input parameters and analyse the spatiotemporal evolution of the body motion. We introduce the *specific tail amplitude*, defined as the ratio of the tail amplitude to a quarter of a wavelength, and the travelling wave index to classify and characterise the swimming performance of anguilliform swimmers.

## Results

**Figure 1:**
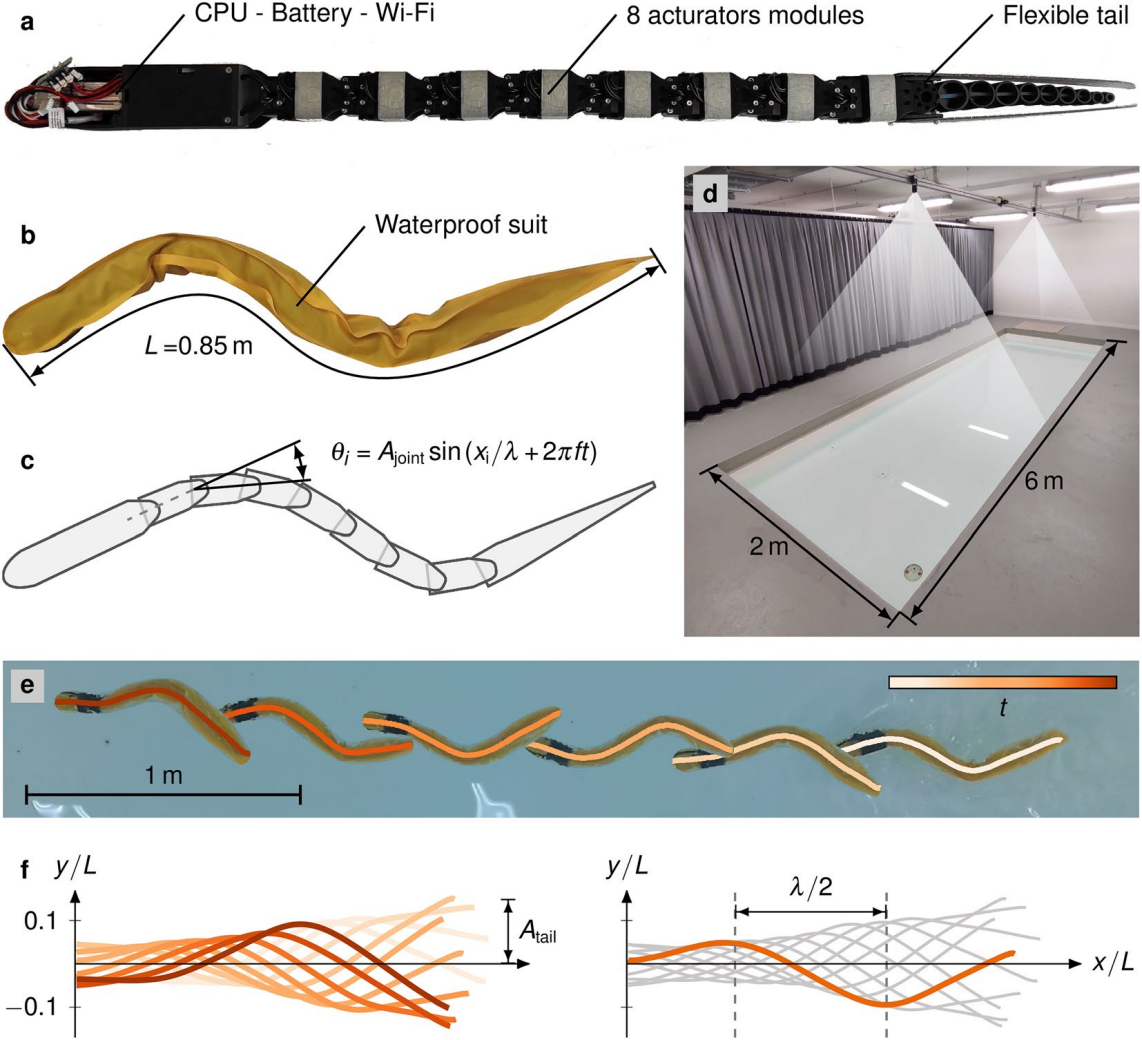
Overview of the experimental set-up. (**a**) Photograph of the anguilliform bio-mimetic robot 1-guilla (pronounced one-guilla or /æŋ’guil/ǝ) that is composed of 8 actuator modules, a flexible tail, and a rigid head that contains the CPU and battery for controlling the undulatory body motion. (**b**) Photograph of 1-guilla with the waterproof suit used during experiments. The full body length (*L*) is used as a characteristic length scale for non-dimensionalisation. (**c**) Schematic of 1-guilla and definition of the input kinematics. (**d**) Swimming pool with a tracking system. (**e**) Overlaid photographic captures of 1-guilla swimming from right to left for ${5.1}\,\hbox {s}$. Pictures are taken at equal time periods of ${0.85}\,\hbox {s}$. The input kinematics have an input wavelength of ${\lambda }_{\textrm{input}}/L=0.71$, joint amplitude ${A}_{\textrm{joint}}={25}^{\circ }$ and a frequency of $f={1.5}\,\hbox {Hz}$. Midline kinematics are overlaid on the pictures, showing an example of the lab coordinate system results. (**f**) The midline for the same kinematic input projected onto the axis that corresponds to the main direction of swimming. The wavelength is extracted as twice the average projected distance between local peaks in curvature. The tail amplitude is calculated as half the peak-to-peak distance of the tip of the flexible tail normal to the swimming direction.

**Figure 2:**
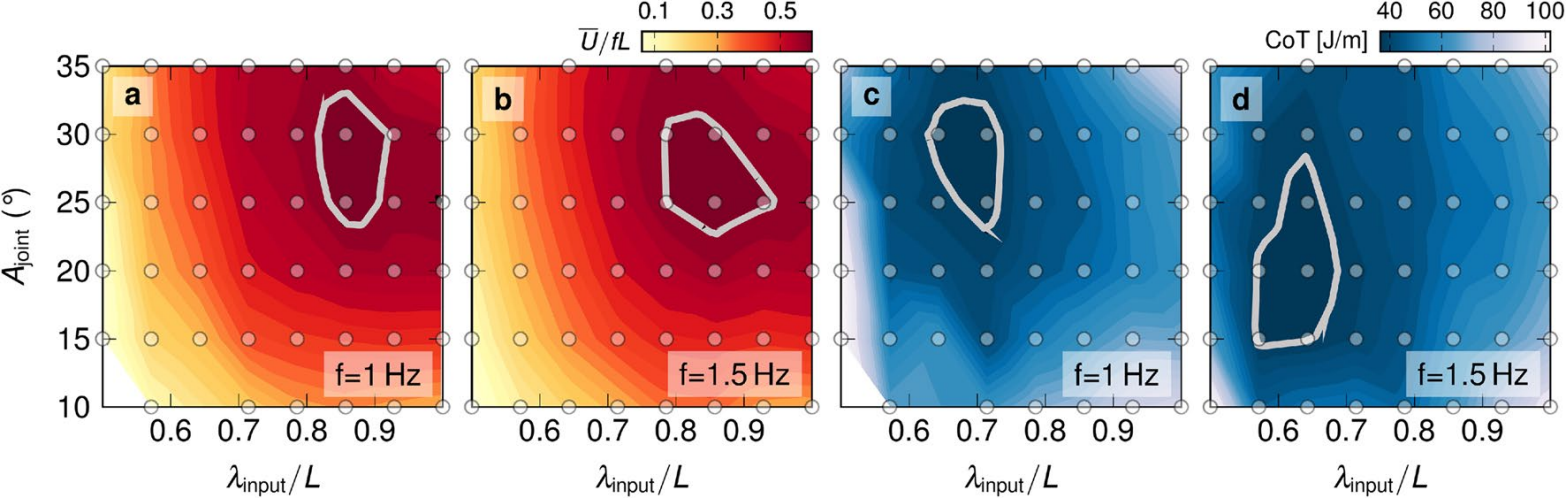
Performance maps. Normalised stride length performance maps (**a**) for $f={1}\,\hbox {Hz}$ and (**b**) for $f={1.5}\,\hbox {Hz}$. Cost of transport performance maps (**c**) for $f={1}\,\hbox {Hz}$ and (**d**) for $f={1.5}\,\hbox {Hz}$. Highlighted contours correspond to results within ${5}\,\%$ of the maximum normalised stride length and the minimum cost of transport, respectively. The circular markers indicate the measurement points.

Figure 1 shows an outline of the robot, testing arena, and data processing procedure. The swimming performance of the robot was characterised through free-swimming experiments in the $6\,{\textrm{m}} \times 2\,{\textrm{m}}\times 0.3\,{\textrm{m}}$ (length × width × depth) pool at EPFL (Fig. 1d). The robot was covered in a waterproof suit (Fig. 1b, supplementary fig. S1). Enough air is trapped in the suit such that the robot floats just below the water surface where it swims freely using open-loop kinematic control. Here, we have focused on kinematics where the joint amplitude is the same for all 8 joints of the robot (Fig. 1a). The prescribed motion is a travelling wave characterised by the joint angle, spatial wavelength, and frequency (Fig. 1c). The joint amplitude ${A}_{\textrm{joint}}$ is varied from ${15}^\circ {\text{ to }} {35}^\circ$ in steps of ${5}^\circ$, the input wavelength ${\lambda }_{\textrm{input}}$ from ${0.5}\,\,\hbox {to}\,{1}\,{L}$ in steps of ${0.071}\,{L}$, and the frequency *f* from ${0.75}\,\hbox {to}\, {3}\,\hbox {Hz}$ in steps of ${0.25}\,\hbox {Hz}$. In total, 152 different input kinematic patterns were tested, and each kinematic pattern was repeated 3 times.

The pool is equipped with an LED tracking system and a video camera to measure the speed and the resulting swimming kinematics (Fig. 1d). From the LED tracking system, we measured the position of a single LED placed on the head of the robot at a frequency of ${15}\,\hbox {Hz}$ and computed the mean steady-state swimming speed $\overline{U}$. The average forward displacement of the robot in terms of body lengths per undulation period, given by $\overline{U}/f$, is called the stride length. Here, we normalise the stride length by the body length *L*, to obtain the normalised stride length $\overline{U}/fL$. The normalised stride length allows a direct comparison of the swimming speed between animals and robots with different body lengths. From the videos, we extract the midline of the robot at a frequency of ${30}\,\hbox {Hz}$. The midline kinematics in the laboratory coordinate system are obtained by image processing the video capture (Fig. 1e, Methods, supplementary fig. S2, and supplementary video S1). Figure 1e shows overlaid images of the robot and the computed midlines at different time steps. We use the lab coordinate system to trace the tail and extract the tail’s amplitude ${A}_{\textrm{tail}}$ as half of the peak-to-peak motion of the tail, including the passive flexible end, normal to the swimming direction (Fig. 1f). For further analysis, the midlines are projected onto the axis that corresponds to the main direction of swimming (Fig. 1f). Based on the projected kinematics, we calculate the performed wavelength $\lambda$, and other performance metrics (see Methods). Additionally, we measure the current *I* and voltage *V* applied by the motors and determine the average power consumption as $\overline{P}=\overline{VI}$. A dimensional cost of transport *CoT* is computed as the ratio of the average power consumption $\overline{P}$ to the average swimming speed $\overline{U}$. More detailed descriptions of the experimental procedure and equipment are provided in the methods section at the end of the paper.

### Performance maps

Example results of the swimming performance of our robot as a function of the non-dimensional input wavelength (${\lambda }_{\textrm{input}}/L$) and the prescribed joint amplitude (${A}_{\textrm{joint}}$) are presented in Fig. 2 for two input frequencies ($f={1}\,\hbox {Hz}$ and $f={1.5}\,\hbox {Hz}$). The contours represent the normalised stride length in Fig. 2a,b and the cost of transport in Fig. 2c,d (see supplementary fig. S3 for contours of the dimensional swimming velocity).

Overall, the stride length can be increased by increasing the wavelength up to ${\lambda }_{\textrm{input}}/L\approx 0.85$ and by increasing the joint amplitude up to ${A}_{\textrm{joint}}\approx {27}^{\circ }$ for both frequencies presented in Fig. 2. A maximum normalised stride length of $\overline{U}/fL = 0.60$ and 0.59 is reached for $f={1}\,\hbox {Hz}$ and ${1.5}\,\hbox {Hz}$, respectively. For the parameter range tested, we find one optimum region where the maximum stride length is reached. We highlight the contour of the regions in the graphs that lie within ${5}\,\%$ of the maximum normalised stride length. For wavelengths above ${\lambda }_{\textrm{input}}/L\approx 0.85$ and joint amplitudes above ${A}_{\textrm{joint}}\approx {27}^\circ$ the stride length decreases. An increase in frequency does not substantially affect the stride length. For an input wavelength ${\lambda }_{\textrm{input}}/L = 0.56$, we varied the frequency from $f={1}\,\hbox { to}\,{3}\,\hbox {Hz}$ in steps of ${0.25}\,\hbox {Hz}$ and measured the normalised stride length. The stride length increases with increasing joint amplitude but remains relatively constant for increasing frequency (supplementary fig. S4). This agrees with previous work showing that speed increases linearly with frequency for undulatory swimming animals^20,21,35^.

The cost of transport also has one optimal region in the parameter space covered. The minimal cost of transport is ${41.1}\,\hbox {J/m}$ for $f={1}\,\hbox {Hz}$ and ${38.1}\,\hbox {J/m}$ for $f={1.5}\,\hbox {Hz}$. We highlight the contours in the cost of transport graphs that bound regions that are within ${5}\,\%$ of the minimum cost of transport. The optimal region for $f={1.5}\,\hbox {Hz}$ lies at a lower wavelength and lower amplitude range than the highlighted contour for $f={1}\,\hbox {Hz}$. The increase in frequency changes the location of the optimum region for the cost of transport, indicating a more complex relationship between the cost of transport and the parameters governing the body kinematics.

The optimal regions for high stride length and low cost of transport do not coincide. Overall, the optimum regions for normalised stride length are found at non-dimensional input wavelengths in the range of ${0.78}\,\hbox {to}\,{0.92}$ and joint amplitudes in the range of ${23}^\circ \,\hbox {to}\,{33}^\circ$. The optimum regions for the cost of transport occur at lower input wavelengths (${0.55}\,\hbox {to}\,{0.71}$) and moderately lower joint amplitudes (${13}^\circ \,\hbox {to}\,{33}^\circ$).

From a control strategy point of view, the motion of an undulatory robot or fish is a compromise between efficiency and speed. Both efficiency and speed depend on the joint amplitude, undulation wavelength, and frequency. For maximum speed, our results suggest that the robot or animal should use higher amplitudes and wavelengths. For efficiency, it should use a lower wavelength and moderate amplitude. These first results suggest that the trade-off between speed and efficiency is more sensitive to wavelength than amplitude. The results here give a practical guide to similar robotic applications. Still, we lack the interpretation of the mechanism that describes the stride length and the cost of transport in response to performed kinematics. The next sections will address these mechanisms by proposing metrics that quantify the impact of the body waveform on the normalised stride length and cost of transport.

### Stride length as a result of the tail motion

**Figure 3:**
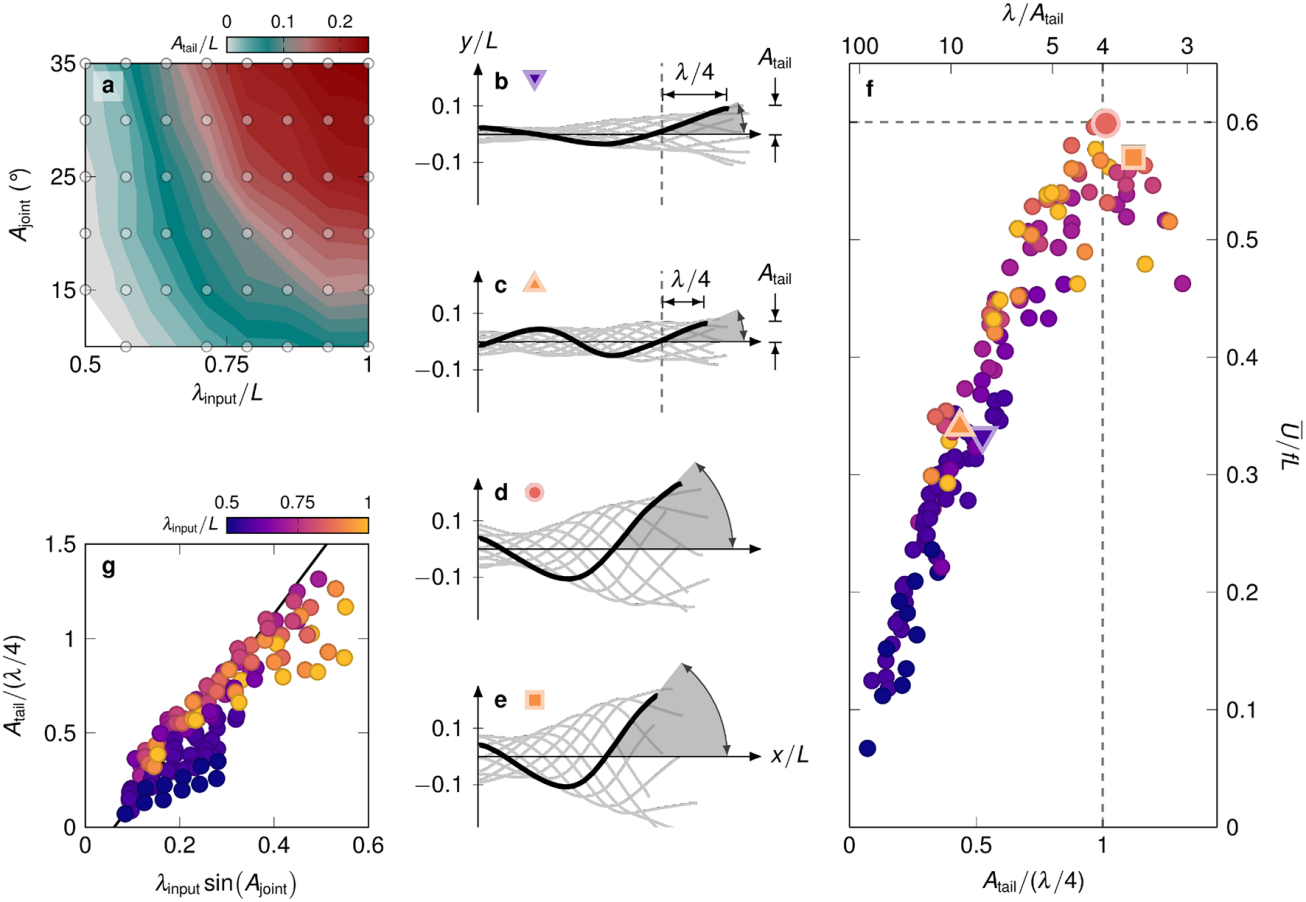
The motion of the tail segment dominates the generation of thrust. (**a**) Tail amplitude ${A}_{\textrm{tail}}$ as a function of ${\lambda }_{\textrm{input}}/L$ and ${A}_{\textrm{joint}}$. The tail amplitude is calculated here as the maximum displacement of the tip of the flexible tail normal to the swimming direction or half of the peak-to-peak distance. (**b**) Midline kinematics for ${\lambda }_{\textrm{input}}=0.57$ and ${A}_{\textrm{joint}}={30}^\circ$. (**c**) Midline kinematics for ${\lambda }_{\textrm{input}}=0.93$ and ${A}_{\textrm{joint}}={10}^\circ$. The kinematics in (**b**) and (**c**) yield a similar normalised stride length and similar specific tail amplitude, for different input parameters. (**d**) Midline kinematics corresponding to optimal normalised stride length for ${\lambda }_{\textrm{input}}=0.85$ and ${A}_{\textrm{joint}}={25}^\circ$ (see supplementary video S2 for the corresponding video). (**e**) Midline kinematics for a specific tail amplitude larger than unity, for ${\lambda }_{\textrm{input}}=0.92$ and ${A}_{\textrm{joint}}={35}^\circ$. The kinematics in (**d**) and (**e**) yield a similar maximum tail amplitude despite different input parameters. (**f**) Normalised stride length $\overline{U}/ f L$ as a function of specific tail amplitude ${A}_{\textrm{tail}}/(\lambda /4)$ for all measurements. The maximum normalised stride length of 0.6 is achieved for the specific tail amplitude of 1. For comparison, the specific wavelength $\lambda /{A}_{\textrm{tail}}$^28,29^ is added as a second x-axis on top for direct comparison (for data plotted directly as a function of the specific wavelength see supplementary fig. S5). (**g**) Specific tail amplitude linearised by input kinematics in the form of ${\lambda }_{\textrm{input}} \sin {({A}_{\textrm{joint}})}$. A linear interpolation of the presented data results in an $R^2$ value of 0.80.

Fishes mostly alter their steady swimming speed by changing their tail beat frequency and show little or no change in amplitude^20,22,36,37^. In general, neither fishes nor robots can directly prescribe the tail amplitude in the laboratory frame; instead, it emerges due to the interaction of the joint amplitudes and the movement of the body’s centre of mass. The tail amplitude is presented in Fig. 3a as a function of joint amplitude and input wavelength for a frequency of ${1}\,\hbox {Hz}$. The tail amplitude increases monotonically by increasing the joint amplitude or the input wavelength to a maximum value of ${A}_{\textrm{tail}}/L=0.277$. We expect that the tail amplitude will keep increasing for further increases in input wavelength and joint amplitude. For our experiments, both performance metrics (stride length and efficiency) decrease for increasing wavelength and amplitude near the maximum values of the parameter space, and a further increase of the parameter space would not be of interest.

To incorporate the dependency of tail amplitude on the input body wavelength and joint amplitude, we define a dimensionless *specific tail amplitude* as the ratio of the tail amplitude to a quarter of the measured body wavelength: ${A}_{\textrm{tail}}/(\lambda /4)$. The quarter wavelength is the distance between the tip of the tail and the most aft location along the body that is at $y/L=0$ when the tip of the tail reaches its maximum value (Fig. 3b–e). The quarter wavelength indicates the length of the active aft portion of the body at the end of the stroke. The tail amplitude only indicates the distance travelled by the tip of the tail normal to the swimming direction. It does not represent the actual motion of the aft of the body, which is not a heaving motion but resembles rather a rotation around the three-quarter wavelength point with an angular amplitude that is best approximated by $atan({A}_{\textrm{tail}}/(\lambda /4))$. The ratio ${A}_{\textrm{tail}}/(\lambda /4)$ is therefore referred to as the specific tail amplitude. It is related to the inverse of the specific wavelength that was introduced in prior studies^28,29^. We prefer to use the specific tail amplitude here instead of the specific wavelength as the values of specific tail amplitude are more directly linked to the tail’s maximum angle at the end of the stroke (supplementary fig. S6).

In Fig. 3b,c, we show examples of two kinematics with similar normalised stride length and specific tail amplitude, but different body waveform. The input parameters for the two points differ substantially: for (b) ${\lambda }_{\textrm{input}}=0.57$ and ${A}_{\textrm{joint}}={30}^\circ$, and for (c) ${\lambda }_{\textrm{input}}=0.86$ and ${A}_{\textrm{joint}}={10}^\circ$. The specific tail amplitude is a measure for the angle that the tail forms with the x-axis at the end of the stride, represented by the shaded arc in Fig. 3b–e. The two example kinematics in Fig. 3b,c yield a similar tail angle as expected based on their similar specific tail amplitude (see also supplementary fig. S6).

The normalised stride length ${\overline{U}}/f {L}$ is presented as a function of the specific tail amplitude in Fig. 3f. For low specific tail amplitudes, the increase in specific tail amplitude results in an increase in the normalised stride length. The slope of this increase is universal across wavelengths and frequencies tested. This universality suggests that the motion of the tail segment dominates the generation of thrust. Other measures such as the tail amplitude and the Strouhal number ($St=2{A}_{\textrm{tail}}f/\overline{U}$) provide a similar prediction of the normalised stride length (supplementary fig. S7). Our robot can reach a maximum normalised stride length of 0.6. The maximum is reached for a specific tail amplitude of $\approx 1$. For specific tail amplitudes above 1, the normalised stride length decreases.

The specific tail amplitude is proportional to the angle between the tail and the direction parallel to the motion. We hypothesise that at low specific tail amplitudes, the lateral motion of the tail is not sufficient to form vortices that transfer enough momentum into the wake and the normalised stride length is low. As the specific tail amplitude increases, we expect the strength of the momentum transferred into the wake to increase and produce more thrust and displacement per cycle, until reaching the maximum normalised stride length of 0.6. The body kinematics that correspond to the maximum normalised stride length are presented in Fig. 3d (see supplementary video S2 for the corresponding video). The respective tail angle at the end of the cycle is $\approx {45}^\circ$. We hypothesise that for specific tail amplitudes $\ge {45}^\circ$, the tail exerts more force on the flow in the lateral direction, i.e. normal to the swimming direction, than in the swimming direction. This leads to a loss in stride length and efficiency (Fig. 3e).

To close the link between the specific tail amplitude and the input kinematic parameters, the variation of the specific tail amplitude is presented in Fig. 3g as a function of the input kinematics: ${\lambda }_{\textrm{input}} \sin {({A}_{\textrm{joint}})}$. This expression of the input kinematics is the simplest expression that results in an approximately linear relationship with the tail amplitude. A linear interpolation of the presented data results in an $R^2$ value of 0.80. The specific tail amplitude monotonically increases with increasing ${\lambda }_{\textrm{input}}$ and ${A}_{\textrm{joint}}$. We observe here that the input wavelength is proportional to the specific tail amplitude for a fixed joint amplitude.

### Efficiency as a result of travelling wave behaviour and whole-body movements

**Figure 4:**
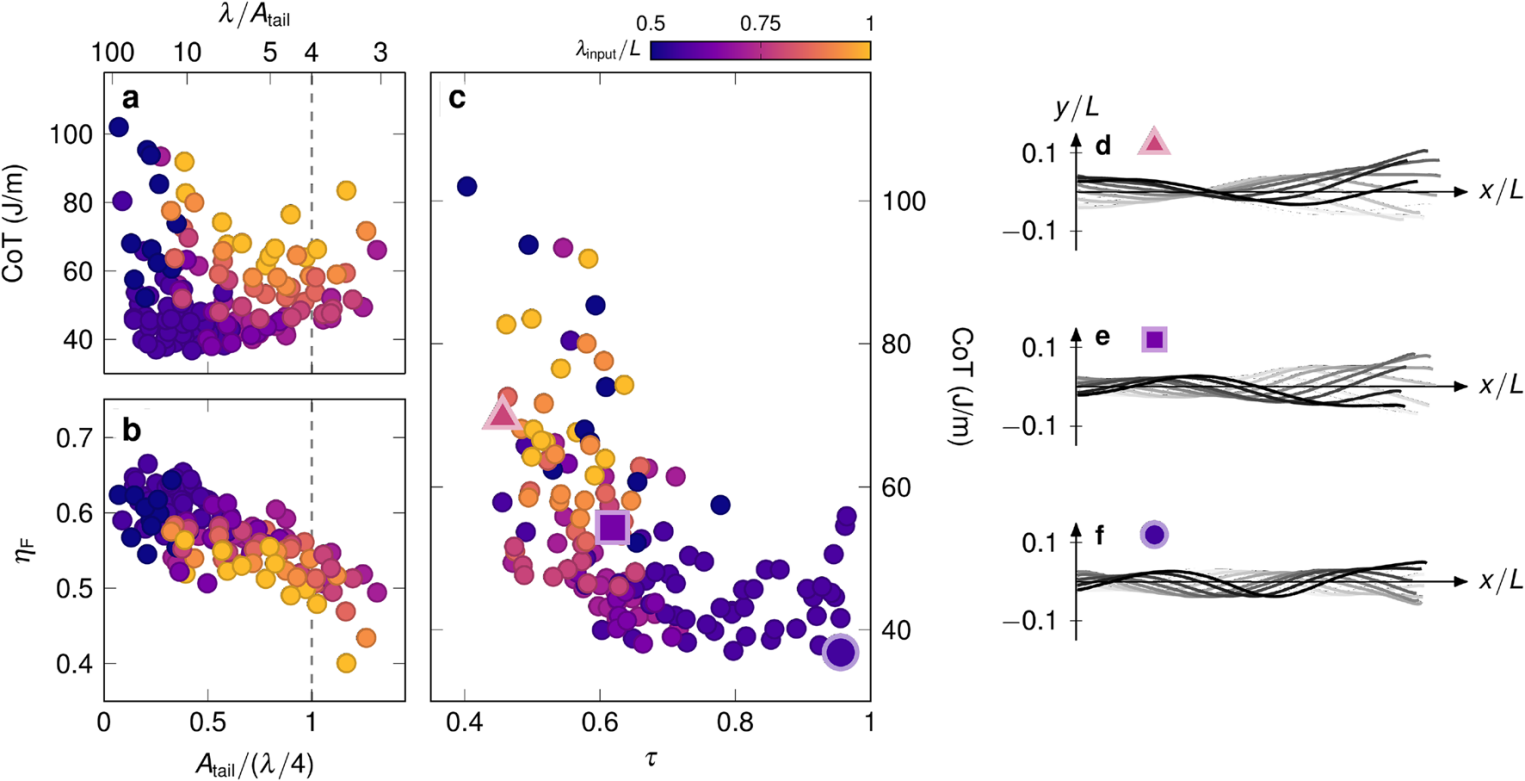
Efficiency as a result of travelling wave behaviour and whole-body movements. (**a**) Cost of transport *CoT* as a function of the specific tail amplitude for all frequencies tested. (**b**) Froude efficiency ${\eta }_{\textrm{F}}$ versus the specific tail amplitude. (**c**) Cost of transport *CoT* versus the travelling wave index $\tau$. Across our results, a higher travelling wave index results in a decrease in the cost of transport. (**d**) Example of a waveform with a low travelling wave index ($\tau = 0.45$), resulting in a high cost of transport. The waveform has a node-like shape, characteristic of standing waves. (**e**) Example of a waveform with an intermediate travelling wave index ($\tau = 0.61$) resulting in an intermediate cost of transport. (**f**) Example of a waveform with a high travelling wave index ($\tau = 0.96$) resulting in the lowest cost of transport (see supplementary video S3 for the corresponding video).

Efficiency in undulatory swimming is expressed by various metrics in the literature. The cost of transport, defined as the ratio between the average power consumed and the average swimming speed, is primarily used by roboticists and biologists that have access to electric or metabolic power consumption^5,38^. For animals, it is one of the most important metrics, because it represents the energy they need to travel over a given distance^4,21,39,40^.

The Froude efficiency is used by tethered robotic applications or simulations where the thrust force is known^18,26,41,42^. If the thrust force is not measured directly, it can be estimated for steady-state swimming using Lighthill’s large-amplitude elongated body theory^43^. Here, we proceed to compare Froude efficiency, estimated through Lighthill’s theory (see Methods), to our cost of transport measurements.

Our measured cost of transport *CoT* is presented as a function of the specific tail amplitude in Fig. 4a. Swimming with a low specific tail amplitude (${0.2}\,{\textrm{to}}\,{0.5}$) can result in the lowest costs of transport. The lowest cost of transport ${CoT}_{\textrm{min}}={36.8}\,\hbox {J/m}$ occurs for a wavelength of $\lambda /L \approx 0.56$ and a specific tail amplitude of 0.4. For a fixed wavelength, intermediate values of specific tail amplitude are associated with a lower cost of transport. For values of specific tail amplitude near unity, where the maximum stride length is achieved, the cost of transport is sub-optimal. This underscores the existence of a trade-off between speed and efficiency identified in Fig. 2. Overall, specific tail amplitude and cost of transport are not well correlated. That suggests that the specific tail amplitude alone (the same holds for the tail amplitude and the Strouhal number as shown in supplementary fig. S7) is insufficient to describe the variation in the cost of transport observed in our results and that the effect of the whole-body movement needs to be considered.

The Froude efficiency ${\eta }_{\textrm{F}}$, as calculated through Lighthill’s large-amplitude elongated body theory, is presented in Fig. 4b versus the specific tail amplitude. Overall, the Froude efficiency decreases with increasing specific tail amplitude and varies from ${0.40}\,{\textrm{to}}\,{0.66}$. The highest Froude efficiency occurs for low specific tail amplitudes (${0.2}\,{\textrm{to}}\,{0.5}$), where the measured cost of transport varies substantially. Lighthill’s theory is based on an inviscid flow approach and favours low-amplitude kinematics. Here, overestimation of efficiency is especially evident for kinematics with low specific tail amplitude. The wake energy waste term ${P}_{\textrm{wake}}$ in Lighthill’s theory (see Methods) is proportional to the tail’s transverse movement, enhancing the efficiency for low specific tail amplitudes. This overestimation provides further evidence that the Froude efficiency is not a reliable substitute for the cost of transport as a metric of efficiency for our experiments^4,39^.

Our results suggest that focusing on tail motion alone does not capture the complexity of the undulatory locomotion, particularly regarding energy consumption. The specific tail amplitude and Lighthill’s theory focus on the tail movement rather than the whole-body motion. In contrast, whole-body motion, specifically travelling-wave kinematics, has been associated with high propulsive efficiencies^41,44,45^. To assess the travelling nature of the swimming kinematics, we conducted a complex orthogonal decomposition (COD)^46,47^. We use the single x-axis projection of our kinematics to perform the COD (Fig. 1f). Through COD, the waveform can be decomposed into dominant complex orthogonal modes, and each mode can be decomposed into its travelling and standing undulatory signals. The decomposition technique offers a metric, called the travelling wave index $\tau$, that quantifies the travelling nature of every mode. A travelling wave index of 1 describes a purely travelling wave, and a travelling wave index of 0 describes a purely standing wave.

To examine the importance of travelling wave kinematics, the cost of transport *CoT* is presented in Fig. 4c versus the travelling wave index $\tau$. The travelling wave index varies from ${0.40}\,{\textrm{to}}\,{0.96}$ across our results, and a higher travelling wave index results in a decrease in the cost of transport. Exemplary kinematics corresponding to low, intermediate, and high travelling wave indices are presented in Fig. 4d–f, respectively. Our results indicate that moderate to small input wavelength ($\lambda <0.6$), higher frequency ($f>\,{1.5}\,\hbox {Hz}$) and moderately low tail amplitudes ($0.05<{A}_{\textrm{tail}}/L<0.1$) result in high travelling wave behaviour (Fig. 4 and supplementary fig. S8). High travelling wave kinematics (Fig. 4f and supplementary video S3) will consistently direct the flow towards the wake with a direction opposing the body motion, creating thrust through momentum flux^44^. A potential explanation for this is that the proto-vortices created near the head will continue to grow, adding momentum to the wake when shed by the tail^14^. The whole-body coordination will then contribute to a high-efficiency motion requiring a low cost of transport. In cases of a low travelling wave index (Fig. 4d), standing wave behaviour will laterally push the surrounding fluid away, without creating a momentum flux in the direction of motion. Proto-vortices created near the head will most probably be shed before reaching the tail, and will not contribute to an exploitable momentum flux in the wake. The momentum flux will happen in directions lateral to the motion resulting in low-efficiency kinematics. A similar mechanism of efficiency is described in the literature using particle image velocimetry data on flexible tapered and untapered heaving panels^18,44,48^. In our results, the relationship between the travelling wave kinematics and cost of transport suggests that efficient propulsion requires proper whole-body coordination (as opposed to only particular tail movements) expressed by a high travelling wave index.

### Comparison to data from swimming animals

**Figure 5:**
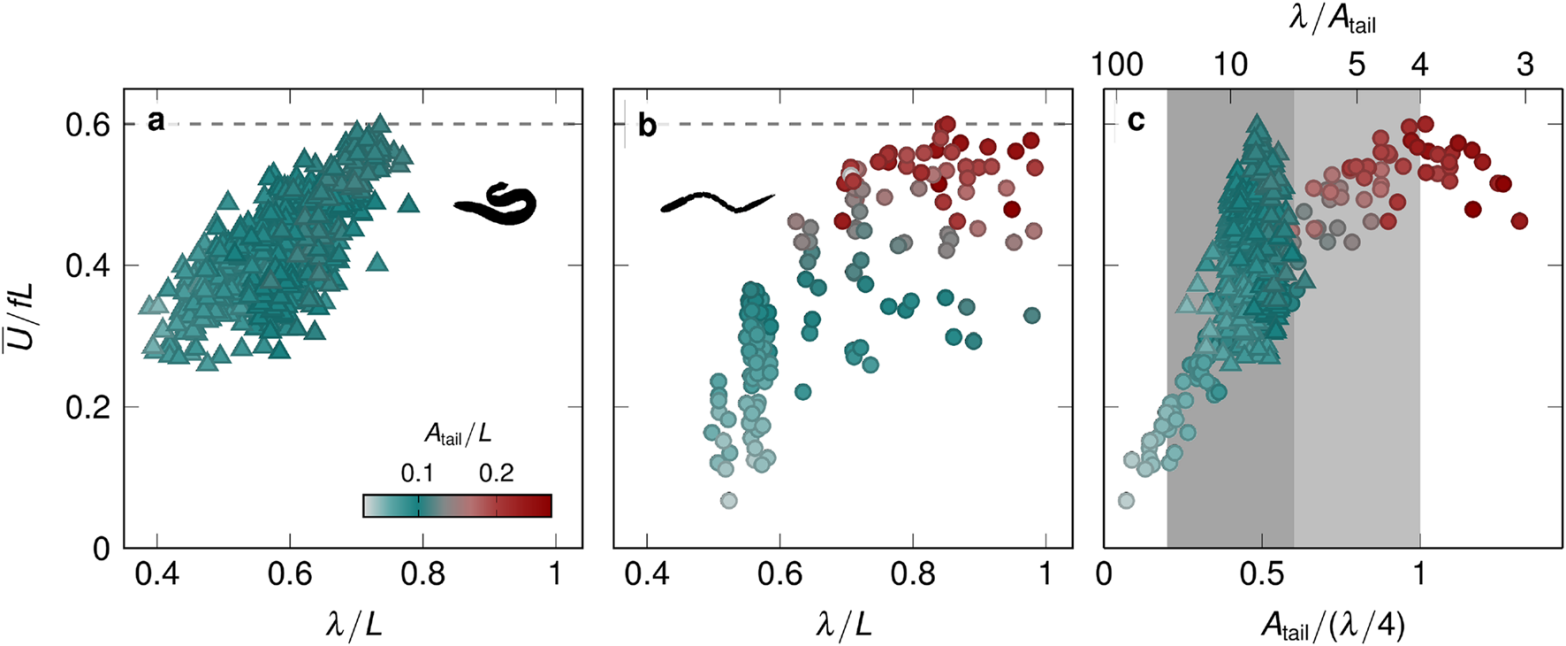
Comparison with data from swimming animals. Normalised stride length versus the measured wavelength for (**a**) eel data from Tytell^22^ and (**b**) for all our experiments with the robot 1-guilla. The individual data points are coloured corresponding to their tail amplitude. (**c**) Normalised stride length versus the specific tail amplitude for 1-guilla (circles) and the eel data (triangles). Four regimes of specific tail amplitudes are distinguished: the low specific tail amplitude regime (${A}_{\textrm{tail}}/(\lambda /4)<0.2$), the efficient regime ($0.2<{A}_{\textrm{tail}}/(\lambda /4)<$0.6; dark gray), the fast regime ($0.6<{A}_{\mathrm{tail|}}/(\lambda /4)<$1; light gray), and the overworking regime ($1<{A}_{\textrm{tail}}/(\lambda /4)$).

In Fig. 5a,b we compare the swimming performances of our robot and living eel observations (data from^22^). The normalised stride length is presented versus the measured body undulation wavelength. The eel data^22^ includes measurements for five individual American eels *Anguilla rostrata*, with body lengths ranging from $L={12}\,\hbox {cm to}\,{24}\,\hbox {cm}$, swimming in a water channel with constant flow speeds ranging from $\overline{U}={0.5}\,{L}/\hbox {s to}\,{2}\,{L}/{\textrm{s}}$. In Fig. 5, we colour the points according to the average tail amplitude. The eels show tail amplitudes in the range of ${0.03}\,\hbox {to}\,{0.1}\,{L}$. With our robot, we explored higher tail amplitudes up to ${0.27}\,{L}$. The body wavelength is traditionally considered a relatively fixed parameter for swimming fishes^12,21^, but it actually varies substantially for the swimming eels, from about ${0.4}\,{L}\,\hbox {to}\,{0.75}\,{L}$. Unlike many other swimming robots, 1-guilla achieves normalised stride lengths up to 0.6, in the same range as those of swimming eels^22^ (Fig. 5). The normalised stride length increases with increasing wavelength for our robot and the eels. For our robot, the increase in normalised stride length is a result of the increase in the specific tail amplitude, which is linearly proportional to the input wavelength. For eels, normalised stride length increases mainly with increasing wavelength. The eels achieve equal or higher normalised stride lengths than our robot for the same undulation wavelengths. This might be due to a combination of their flexibility and a more complicated modulation of the body waveform. For this study, we have used a constant amplitude for all joints of our robot, which leads to a constant curvature along the body, whereas eels and other fishes typically display an increasing curvature along the body^22^. Our robot has a passively flexible tail and the tail amplitude does not continuously increase with increasing wavelength, whereas the eels can actively stiffen their tail to continuously increase the tail amplitude with wavelength^32,49,50^. Overall, the eels show an increased wavelength when their stride length increases, perhaps indicating a similar trade-off between swimming speed and swimming efficiency as we saw in our robot data.

## Discussion

In this paper, we analysed the trade-off between speed and cost of transport for a bio-inspired anguilliform robot for a wide range of the undulation wavelengths, joint amplitudes, and frequencies, which are the main kinematic parameters that control the body undulation. We reanalysed swimming data from American eels, *A. rostrata*^22^, and found similar patterns. Performance maps indicate that for maximum speed, the robot or animal should use higher amplitudes and wavelengths. For efficiency, it should use a lower wavelength and moderate amplitude. Midline kinematics shed further light on the speed and efficiency trade-off. We introduced the non-dimensional specific tail amplitude defined as the ratio of the tail amplitude to a quarter of a wavelength as a measure of the angle that the tail forms with the swimming direction at the end of a stride. Different performance regions are distinguished based on the specific tail amplitude.

For our robot, we identified four different swimming regimes as a function of the specific tail amplitude (Fig. 5c). The low specific tail amplitude regime, for specific tail amplitude ${A}_{\textrm{tail}}/(\lambda /4)<0.2$, results in a motion with a relatively high cost of transport and low normalised stride length. The efficient regime, for specific tail amplitude ${A}_{\textrm{tail}}/(\lambda /4) ={0.2}\,{\textrm{to}}\,{0.6}$, is where we observe the lowest cost of transport, but there is a trade-off. The normalised stride lengths achieved in this regime are below ${75}\,\%$ of the maximally achievable normalised stride length of 0.6. To achieve the lowest cost of transport, travelling-wave-like kinematics are required, but with high travelling-wave indices, the resulting tail amplitude tends to be lower, producing a lower normalised stride length. In the fast regime, for specific tail amplitude ${A}_{\textrm{tail}}/(\lambda /4) ={0.6}\,{\textrm{to}}\,{1}$, the normalised stride length increases with increasing specific tail amplitude until reaching the maximum normalised stride length of 0.6. The average cost of transport in this regime increases with increasing specific tail amplitude. Finally, in the overworking regime, for specific tail amplitude ${A}_{\textrm{tail}}/(\lambda /4)>1$, the cost of transport increases rapidly and the normalised stride length decreases with increasing specific tail amplitude.

The specific tail amplitude presented here is four times the inverse specific wavelength proposed by previous studies^28,29^. The values of specific wavelength that were associated with optimal thrust creation based on numerical simulations and robotic sheet experiments ranged from $\lambda /{A}_{\textrm{tail}}={5}\,{\textrm{to}}\,{30}$ for undulating bodies^28^. The values of the specific wavelength observed for steady-swimming body and/or caudal fin swimmers ranged from ${4}\,{\textrm{to}}\,{15}$. Interestingly, most anguilliform swimmers were found in the lower part of the range and swim at specific wavelengths below 10. Both the best performing eel and robot data fall within this broad range of optimal specific wavelengths identified (Fig. 5c). We chose to use the specific tail amplitude rather than the specific wavelength as the non-dimensional stride length varies approximately linearly with the specific tail amplitude. Furthermore, the values of specific tail amplitude are more directly linked to the tail’s maximum angle at the end of the stroke (supplementary fig. S6).

The region of high efficiency and low cost of transport is different from the high normalised stride length and high thrust region. The prerequisite to achieving high efficiency is to adopt pure travelling wave-like body undulations. The identified regimes suggest that the specific tail amplitude acts as a switch in the trade-off between efficiency and speed.

Our results suggest that changing the frequency is good practice to modulate swimming speed while keeping efficient locomotion. As the mapping of the specific tail amplitude suggests, stride length increases with increasing specific tail amplitude but efficient locomotion is obtained at low specific tail amplitudes. For an anguilliform swimmer to modulate its speed while maintaining high efficiency, it is beneficial to keep a low specific tail amplitude with a low stride length, maintain travelling wave kinematics and modulate its tail beat frequency to move faster. The high stride length should be used in cases where high acceleration is needed, such as when escaping a predator or during agile manoeuvring, where power efficiency is not the primary goal. For robotic applications where the structural integrity of the robot might be endangered by a high frequency, our results suggest that a higher specific tail amplitude should be used to achieve higher swimming velocities.

The range of eel data is presented versus the specific tail amplitude in Fig. 5c where the four swimming regimes of our robot are indicated. Eels seem to maintain a constant specific tail amplitude $\overline{{A}_{\textrm{tail}}/(\lambda /4)} = 0.47 \pm 0.05$, which is within the identified efficient specific tail amplitude regime of our robot. In general, fishes are thought to modulate swimming speed primarily by changing tail beat frequency, keeping tail beat amplitude and body wavelength nearly constant^12,20,21^. Our reanalysis of the eel data from Tytell^22^ suggests that variation in body wavelength may be larger than previously expected, and may contribute to swimming performance. Specifically, it appears that eels may vary both wavelength and amplitude to keep a constant specific tail amplitude in the efficient region of specific tail amplitudes. Eels might also use additional mechanisms to achieve higher stride length at lower specific tail amplitude, including an increase in the amplitude envelope along the length of the body^11^, or an increase of muscle activity^50^ that may alter effective body stiffness^51^. Our results suggest a mechanism that anguilliform swimmers might also use by varying their wavelength to achieve a similar transition from efficient to fast kinematics as shown by our robot. The eel experimental data included here was collected for eels swimming on the bottom of a flow channel. Potential differences in swimming performance for swimmers at the bottom, the surface, or in the centre of a flow channel remains a topic for future investigations.

This paper addresses the effect of undulatory kinematics on the swimming performance of anguilliform swimmers. Our results reveal the existence of a trade-off between efficiency and speed. We provide a data-driven guide that an undulatory swimmer has to follow to achieve either high speed or efficiency. Our results tie in well with the concept that robotics and biology can be mutually beneficial^52^. Our robot design is inspired by natural swimmers and has similar performance results. The access to a wide range of kinematics as well as power consumption data allowed us to further analyse undulatory swimming from a new perspective and provide possible explanations for the kinematic choices of natural swimmers even though the efficiency measures of electrical motors and animals differ. We expect the observed trends to be valid as long as the hydrodynamic forces dominate the energy expenditure of living animals. Finally, for future bio-inspired robotic applications, our results enhance our understanding of efficient and robust robot designs and provide guidelines for their control.

## Methods

### Robot design

We use a bio-mimetic anguilliform swimming robot named 1-guilla, inspired by European eels: *Anguilla anguilla*. 1-guilla is a modular robot that consists of 8 active modules, a head unit for computation, and a passively deforming flexible tail that mimics the shape of an eel (Fig. 1a, supplementary fig. S1). Its body length is ${85}\,\hbox {cm}$, similar to the body length of a European eel^13^, and weighs ${1.4}\,\hbox {kg}$, double the weight of its natural counterpart. Each segment of the robot is actuated by a Dynamixel XM430-W210-R servomotor (Robotis). Joints are connected to each other through rigid 3D-printed pieces, directly attached to the shaft of the first motor and the body of the following one. The first joint, which represents the head of the robot, contains a Li-Po battery (nominal voltage of ${11.1}\,\hbox {V}$, capacity ${1500}\,\hbox {mA h}$) and a computational unit. The computational unit of the robot contains a quad-core Raspberry Pi zero 2 W computer running Linux. Through Wi-Fi, we communicate between the robot’s computer and an external PC that controls the timing of each experimental measurement. The robot’s computer is responsible for sending the desired angle value information at a time step of ${20}\,\hbox {ms}$. An internal PID controller drives every motor in a position control mode. The onboard controller unit logs the angular position from all the motors’ encoders, the voltage, and the current consumption of the motors for post-processing. All motors are connected and powered in series through a multi-drop bus following the *RS485* protocol. 1-guilla is equipped with a swimsuit to make it waterproof. Air trapped in the swimsuit lets the robot float just below the water surface. The robot is immersed by ${60}\,\%$ of its height. The suit material is yellow rip-stop fabric coated with a thermo-adhesive material. The seams of the suit were sealed using heat and high pressure (heat press), where the thermo-adhesive material glued the parts together in the process. The suit is equipped with a waterproof zipper (TIZIP, Master-Seal 10, ${500}\,\hbox {mbar}$ pressure proof).

### Experiments

Free-swimming experiments were carried out in the ${6\,\hbox {m}\times 2\,\hbox {m}\times 0.3}\,\hbox {m}$ (length × width × depth) pool at EPFL. The experiment starts by manually setting the initial posture of the robot. We place the robot at the midpoint of one edge of the pool. Before the robot starts the motion we ensure that its body is placed parallel to the length of the pool. To account for slight differences in the initial conditions between experiments, we repeat experiments at least three times. Slight asymmetries between the robot and the initial conditions may lead to the robot swimming in a diverging trajectory. Diverging trajectories where the robot hit the wall before travelling half the length of the pool were rejected.

### Kinematics and motion tracking

Prescribed kinematics follow a simple travelling waveform:

1 $$\begin{aligned} {\theta }_{\textrm{i}}={A}_{\textrm{joint}} \sin {({x}_{\textrm{i}}/{\lambda }_{\textrm{input}}+2\pi ft)} \end{aligned}$$

where ${\theta }_{\textrm{i}}$ is the undulation angle control input for the *i*th joint, starting from the head. ${A}_{\textrm{joint}}$, *f*, and ${\lambda }_{\textrm{input}}$ are the input joint amplitude, frequency, and wavelength.

To track the position and calculate the speed of our robot, we used a dual-camera tracking system (Basler A622F) mounted ${2.25}\,\hbox {m}$ above the pool surface. The cameras track an LED mounted on the head of the robot (Fig. 1 d). A custom script and a calibration tool convert an LED’s position to spatial measurements for an accuracy of less than ${1}\,\hbox {cm}$. The LED was placed on the head of the robot. The system has the capacity to track LEDs at a frame rate of ${15}\,\hbox {Hz}$.

To calculate the steady-state swimming speed of the motion, we isolate a spatial region of steady-state swimming. The spatial region starts ${2}\,\hbox {m}$ from the starting edge of the pool to account for acceleration and ensures a distance of ${20}\,\hbox {cm}$ from the walls. We consider for the calculation the last 5 periods available in the spatial region before the robot hits the boundary of the pool. We fit a 2nd-degree polynomial to the head’s isolated steady-state curve, which represents the trajectory of the robot. Finally, we calculate the average speed by dividing the length of the trajectory by the time period. This method is robust to motions that slightly diverge from a straight-line trajectory.

### Image processing

Midline kinematics are extracted using video footage taken during the experiments. We added a camera (Yi 4K Action Camera) mounted ${2.25}\,\hbox {m}$ above the pool and perform a series of image processing techniques on each frame of the video. The steps of the image processing are presented in supplementary fig. S2.

The first step is to extract the edge of the robot’s shape and convert it into a binary image. Because the pool colour consists of a light blue palette, we can use a simple threshold of the blue channel $\hbox {B}<{100}$ (based on a standard RGB colour scale, each channel’s brightness ranging from 0 to 256) to obtain an initial binary image. The rough edges of the binary image are smoothed in a two steps process. In the first step, the binary image is blurred by applying a homogeneous convolution filter of a ${35}\,\hbox {px}$ window size. The image is now a grey-scale image (each pixel has a ranging brightness intensity from ${0} {\textrm{~to~}} {1}$, with 0 being black). The second step is a brightness intensity threshold of 0.45 to obtain the final binary image. The size of the convolution filter window and the intensity threshold are the results of a tuning process. The selection preserves the geometry of the robot’s shape and excludes artificial edges generated by the initial colour threshold. Finally, the midline is extracted from the binary image by averaging the values of the robot’s body lateral sides that correspond to the same longitudinal coordinate.

#### Projection onto the axis that corresponds to the main direction of swimming

To project each posture onto a single x-axis and account for non-straight trajectories, we fit a 2nd-degree polynomial to all the deformation points of all recorded midlines during five periods of motion. The fitting captures the average trajectory of all midlines considered. For every midline, the single-axis lateral displacement is calculated as the orthogonal distance to the fitted polynomial.

### Power consumption

To measure each motor’s electric power consumption, we average the instantaneous power consumption over five periods of motion. Every motor has an internal power acquisition module that can measure the voltage *V* and current *I* provided. We calculate the average power $\overline{P}$ as the mean product of instantaneous current and voltage: $\overline{P}=\overline{V \cdot I}$. Example raw signals of power consumption for the individual motors are presented in supplementary fig. S9. To ensure that we can trust the data provided by the motor’s power acquisition module, we calculate the time-synchronous signal average of instantaneous power. The standard deviation band of the time-synchronous signal average power remains below $\pm {0.5}\,\hbox {W}$, providing satisfactory accuracy and reliability of the power acquisition module given that the hydrodynamic forces acting on the robot’s body affect each motor’s cycle-to-cycle power consumption. An additional bench test was performed to confirm that the individual motors perform similarly (see supplementary fig. S10). We mechanically decoupled the motors from the shaft and programmed all motors to run the same periodic motion for frequencies ranging from ${1}\,\hbox {to}\,{3}\,\hbox {Hz}$. For all frequencies tested, the standard deviation of the individual motor power consumption remained less than ${7.5}\,\%$ of the average power consumption. The power measurement accounts only for power consumed in the motors. To ensure the correct comparison between different experiments we frequently recharged the battery when its voltage dropped under a specific voltage threshold, well above the recommended minimum.

### Metrics

#### Tail amplitude

Tail amplitude is calculated for every swimming kinematic using the lab coordinate system accounting for five periods of motion. The tail’s trace on a lab coordinate system is first fitted by a second-degree polynomial. The polynomial is used to calculate the orthogonal distance of the trace of the tail, which is then phase-averaged over the five cycles considered. The phase averaging results in the average tail amplitude and standard deviation for every swimming kinematic.

#### Wavelength

The performed wavelength, which may be different from the input wavelength, is calculated for every recorded swimming motion using the single-axis projection coordinate system of the midline. For every swimming kinematic, five periods are used to calculate the average performed wavelength. Half the wavelength is estimated from the projected distance of two consecutive peaks in the body curvature (Fig. 1f).

#### Froude efficiency

To calculate Lighthill’s expression of the Froude efficiency, we follow the methodology presented in the study of the large amplitude elongated body motion^43^. We use the lab coordinate system to calculate Froude efficiency, averaging over five cycles of swimming. Lighthill’s theory defines a Cartesian lab coordinate system (*x,y*), where x is the direction of swimming and y is normal to it. Every cross-section of the body has a lateral velocity $\partial {y}/\partial {t}$. Additionally, the theory defines a body-attached curvilinear coordinate *s*, where $s=0$ for the head and $s=L$ for the tail. The body-attached coordinate system describes the kinematics of the body as *x*(*s*, *t*), *y*(*s*, *t*). Finally, two velocities are defined: (*u*, *w*) as the parallel and the orthogonal velocities to the local tangential direction of the body. Lighthill uses an inviscid fluid flow consideration and vortex shedding arguments to define the average thrust $\overline{T}$ and energy dissipation in the wake $\overline{E}$ per unit of mass as:

2 $$\begin{aligned} \overline{T}=\left( \overline{ w(\partial {y}/\partial {t}-1/2w\partial {x}/\partial {s} }\right) _{s=L}, \overline{E}=\left( \overline{1/2 w^2u }\right) _{s=L}. \end{aligned}$$

Finally, Froude’s efficiency is calculated as:

3 $$\begin{aligned} {\eta }_{\textrm{F}}=\frac{\overline{T}\overline{U}}{\overline{T}\overline{U}+\overline{E}}. \end{aligned}$$

#### Travelling wave index

To perform the complex orthogonal decomposition, we follow the methodology presented by Feeny^46^. We use the single-axis projection of the midline and five periods of swimming. The lateral displacement values are stacked to form a matrix ${Y}_{\text{M} \times \text{N}}$ for *M* evenly distributed points along the length of the robot’s body tracked for *N* time steps. A complex matrix *Z* is constructed using the Hilbert transform $\mathscr {H}$ to add a complex component: $Z=Y+i\mathscr {H}(Y)$. The complex correlation matrix *R* is calculated using *Z*: $R = Z\overline{Z}^T/N$. We then perform a singular value decomposition of the complex correlation matrix $R = V {\Sigma }_{\textrm{R}} \bar{V}^{T}$ that gives complex singular vectors ${V}_{\text{M} \times \text{N}}$ and complex singular values of *R* as the diagonal of ${\Sigma }_{\textrm{R}}$. The columns of *V* represent the complex orthogonal modes, and the diagonal values of $\Sigma_{\textrm{R}}$ are the singular values of *R* capturing the energy of every mode. The first two modes capture ${97.0(3)}\,\%$ of the signal energy. For kinematics where the trajectory is curving significantly, the first mode captures the turning motion and the second mode captures the travelling wave behaviour. This applied to 11 out of 152 measurements. To compute the travelling index of a mode *j*, we construct a two-column matrix with the real and imaginary parts of the $j^\text {th}$ column of *V*, ${v}_{\textrm{j}}$ as ${W}_{\textrm{j}} = \left[ \text {Re}\left( {v}_{\textrm{j}}\right) ^\textsf{T}~ \text {Im}\left( {v}_{\textrm{j}}\right) ^\textsf{T} \right]$. The travelling wave index results as the inverse condition number of ${W}_{\textrm{j}}$: $\tau = \text {Cond}\left( {W}_{\textrm{j}}\right) ^{-1}$.

### Supplementary Information

Supplementary Information.

Supplementary Video S1.

Supplementary Video S2.

Supplementary Video S3.

## Data Availability

All data available in this paper are available upon request by contacting Karen Mulleners (karen.mulleners@epfl.ch).

## Code availability

All the relevant code used to generate the results in this paper is available upon request by contacting Karen Mulleners (karen.mulleners@epfl.ch).
