# Supplementary material for: Identification of the trade-off between speed and efficiency in undulatory swimming using a bio-inspired robot
Source: Sci Rep. 2023 Sep 12;13:15032. doi: 10.1038/s41598-023-41074-9 (PMC10497532; doi:10.1038/s41598-023-41074-9)
Supplement: Supplementary file 1 — Supplementary Information. [file 41598_2023_41074_MOESM1_ESM.pdf]

Supplementary information: Identification of the trade-off between speed and efficiency in undulatory swimming using a bio-inspired robot

Alexandros Anastasiadis[1,2], Laura Paez[2], Kamilo Melo[3], Eric D. Tytell[4], Auke J. Ijspeert[2], and Karen Mulleners[1]

[1] *Unsteady Flow Diagnostics Laboratory, Institute of Mechanical Engineering, École Polytechnique Fédérale de Lausanne (EPFL), Lausanne, CH-1015, Switzerland*
[2] *Biorobotics Laboratory, Institute of Bioengineering, École Polytechnique Fédérale de Lausanne (EPFL), Lausanne, CH-1015, Switzerland*
[3] *KM-RoBoTa, Renens, CH-1020, Switzerland*
[4] *Department of Biology, Tufts University, Medford, MA 02155, USA*

Supplementary Videos

Supplementary Video 1 shows the raw footage of the robot swimming with the input kinematic parameters ($\lambda_{\text{input}}/L = 0.71$, $f = 1.5$ Hz and $A_{\text{joint}} = 25°$) used to explain the kinematics extraction of fig. 1 of the manuscript.

Supplementary Video 2 shows the raw footage of the robot swimming with the input kinematic parameters ($\lambda_{\text{input}}/L = 0.85$, $f = 1$ Hz and $A_{\text{joint}} = 25°$) that result in the maximum normalised stride length of 0.6.

Supplementary Video 3 shows the raw footage of the robot swimming with the input kinematic parameters ($\lambda_{\text{input}}/L = 0.56$, $f = 2$ Hz and $A_{\text{joint}} = 20°$) that result in the minimum cost of transport.

Supplementary Figures

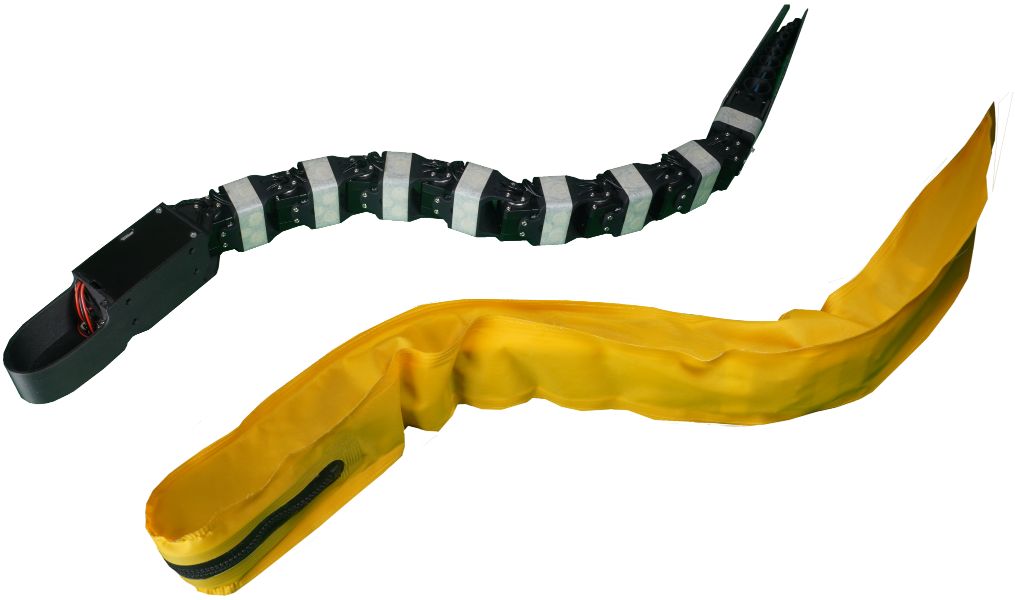

Supplementary Figure S1. Bio-inspired robot 1-guilla without (above) and with its waterproof swimming suit (below).

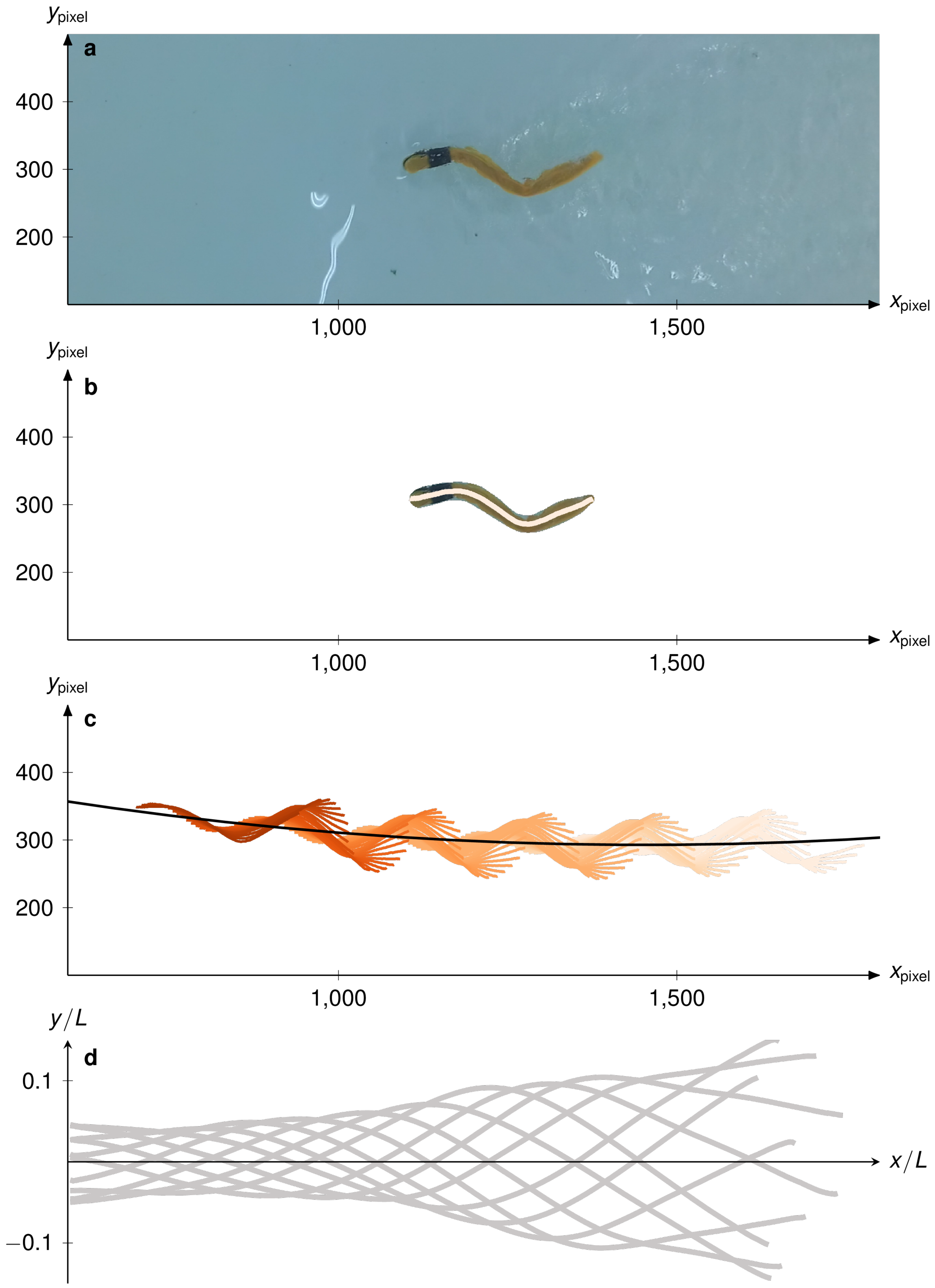

Supplementary Figure S2. Image analysis to kinematics extraction. **a** Raw video footage capture for the kinematics presented in Fig. 1f of the main article. **b** The edge of the robot's shape is extracted and converted into a binary image. A series of image processing techniques is used to extract the edge of the robot (see Methods section of the main article). The midline is extracted from the binary image by averaging the values of the robot's body lateral sides that correspond to the same longitudinal coordinate. **c** The midlines extracted for five periods of motion are plotted and coloured by time. To project each posture onto a single x-axis and account for non-straight trajectories, we fit a 2nd-degree polynomial to all the deformation points of all recorded midlines (black curve). The fitting captures the average trajectory of all midlines considered. **d** The resulting kinematics (same as Fig. 1f of the main text) projected onto the axis that corresponds to the main direction of swimming

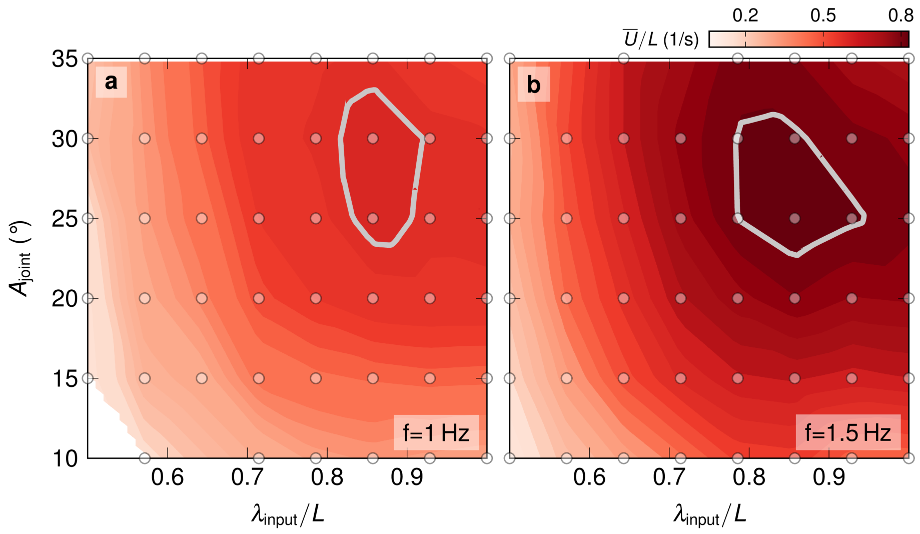

Supplementary Figure S3. Performance maps in terms of velocities, **a** for 1 Hz and **b** for 1.5 Hz. Highlighted contours correspond to results within 5 % of the maximum normalised stride length and the minimum cost of transport, respectively. The circular markers indicate the measurement points.

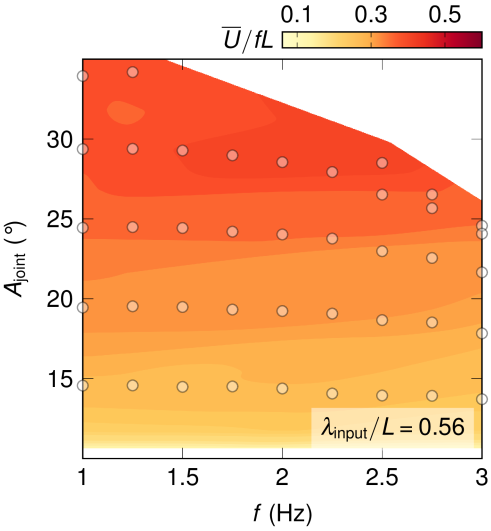

Supplementary Figure S4. Effect of frequency on stride length for a constant wavelength. Normalised stride length as a function of frequency and joint amplitude for constant input wavelength $\lambda_{input}/L = 0.56$. Frequency varies from $f = 1$ Hz to 3 Hz, the normalised stride length increases with increasing joint amplitude but remains relatively constant for increasing frequency. The circular markers indicate the measurement points. For higher frequencies, the robot controller was not capable of producing the requested joint amplitude. The presented joint amplitude is the average of the true joint amplitude recorded from the motor encoders.

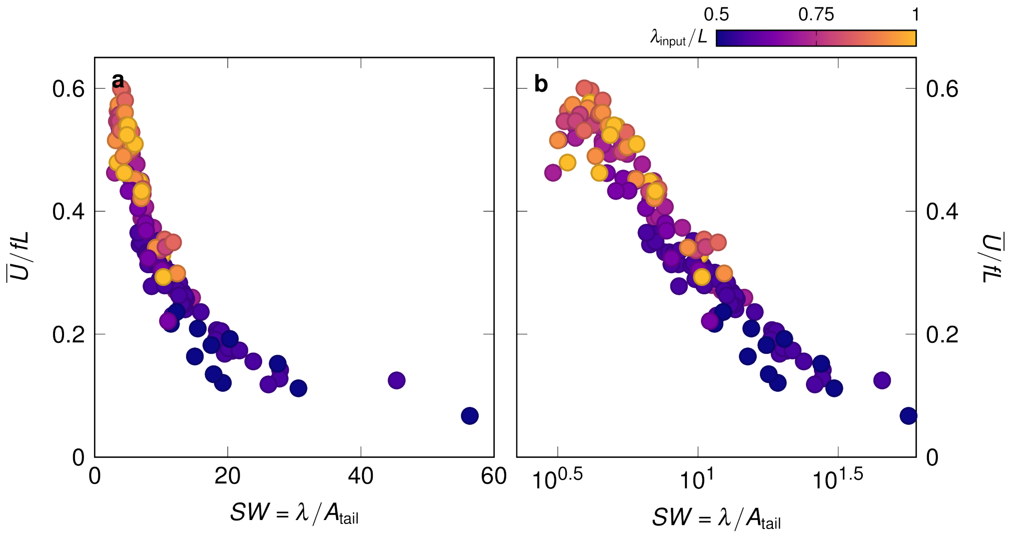

Supplementary Figure S5. Stride length as a function of specific wavelength, with **a** linear axes, and **b** logarithmic axes.

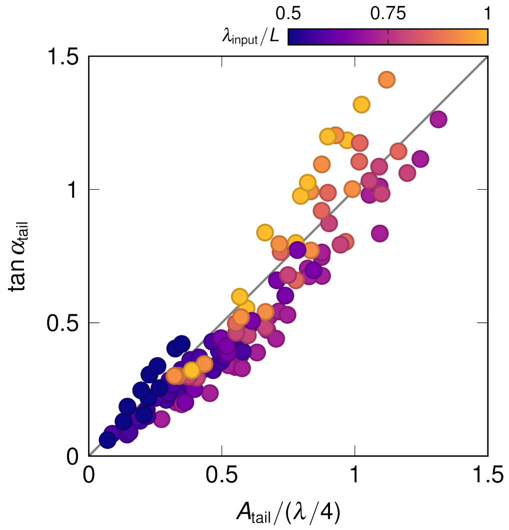

Supplementary Figure S6. Tail angle correlates well with the specific tail amplitude. Maximum tail angle is presented as a function of the specific tail amplitude. A linear regression with the diagonal $\tan \alpha_{\text{tail}} = A_{\text{tail}}/(\lambda/4)$ gives a value of $R^2 = 0.86$. We calculated the temporal evolution of the tail angle of the kinematics by isolating the last segment of the tail for five periods of the motion. The amplitude of the temporal evolution of the tail angle is an estimate of the maximum tail angle.

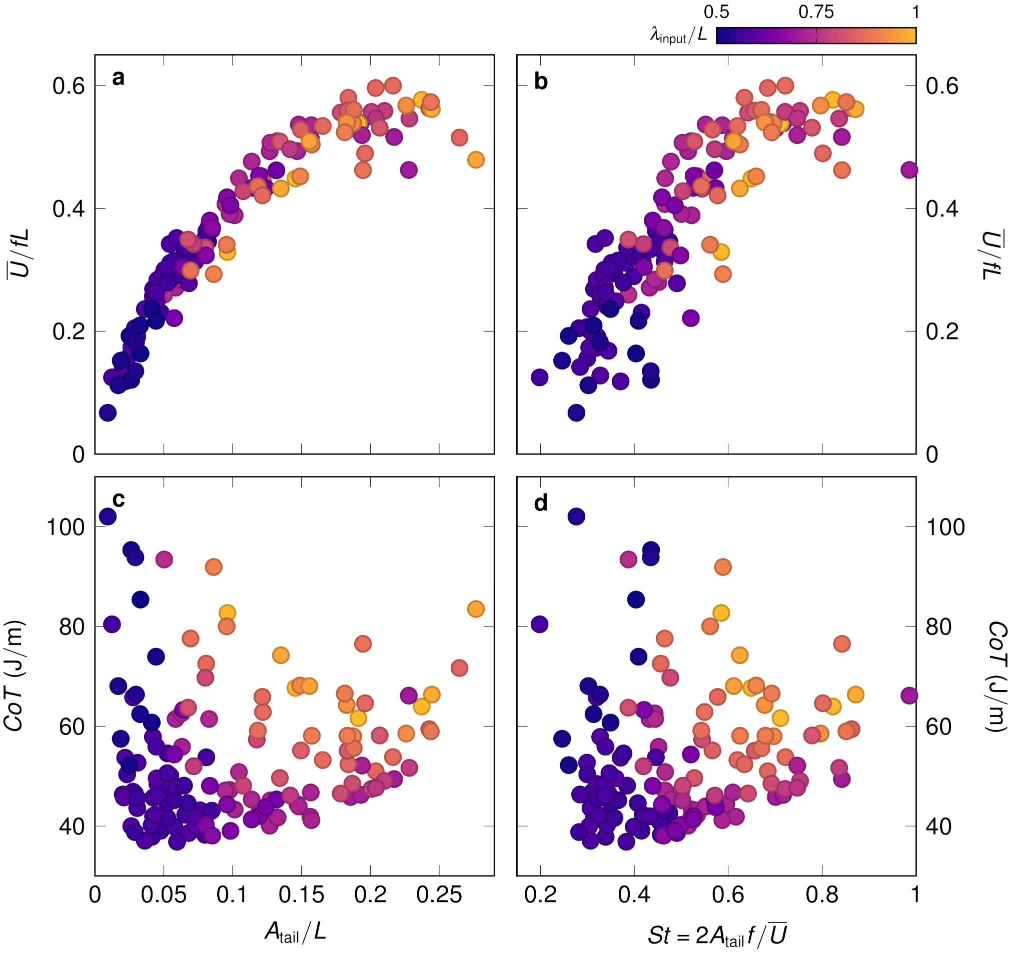

Supplementary Figure S7. Swimming performance metrics as a function of tail amplitude and Strouhal number (*St*). **a** Normalised stride length as a function of tail amplitude. **b** Normalised stride length as a function of Strouhal number. The collapse of the data is worst compared to the tail amplitude and the specific tail amplitude. **c** Cost of transport as a function of tail amplitude. **d** Cost of transport as a function of Strouhal number. Optimum values are present in the optimum region reported in literature as $St = 0.25$ to 0.35. *St* on its own is not sufficient to predict optimal cost of transport.

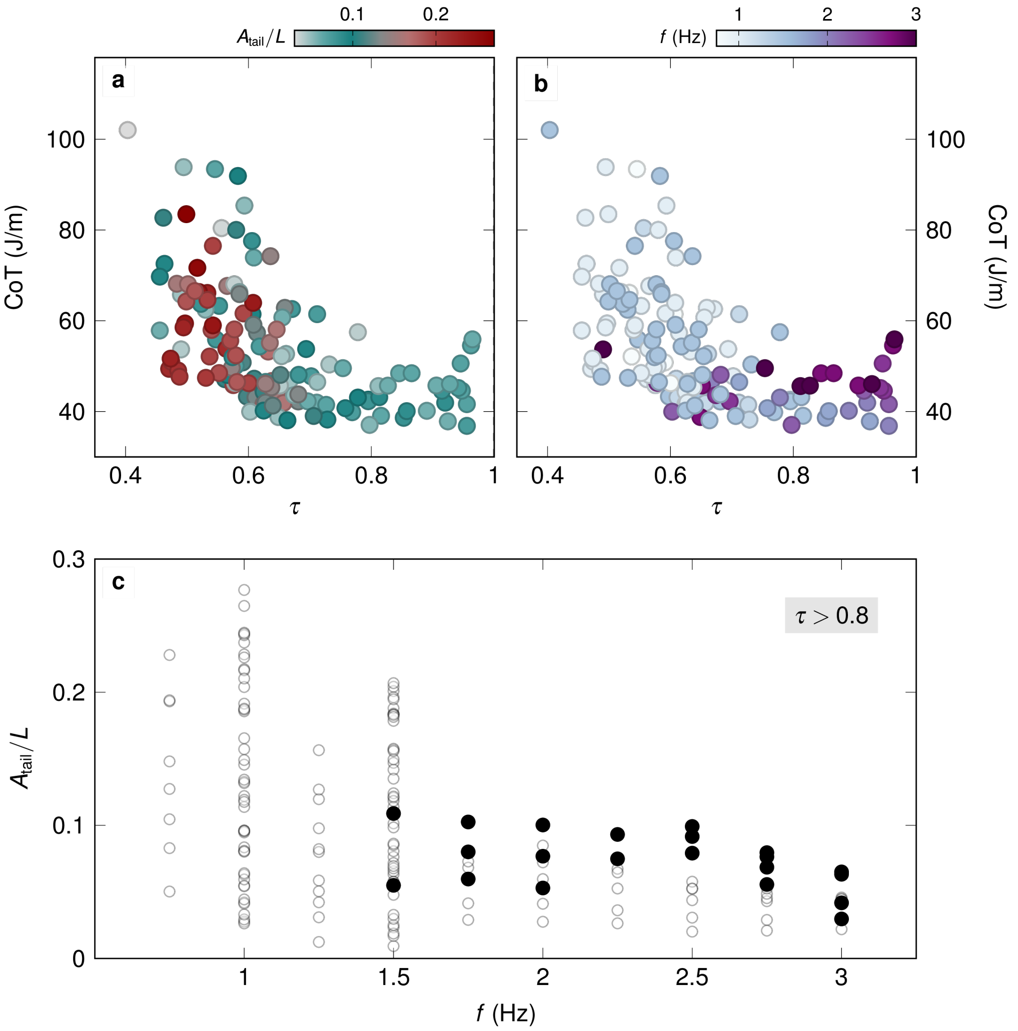

Supplementary Figure S8. How to achieve a high travelling wave index? a Cost of transport CoT versus the travelling wave index τ coloured by the resulting tail amplitude A_{tail}. **b** Cost of transport CoT versus the travelling wave index τ coloured by the input frequency f. **c** Points where the travelling wave index $\tau > 0.8$ in black markers vs all experimental points in white markers, as a function of resulting tail amplitude and input frequency. Moderate to small input wavelength ($\lambda < 0.6$), higher frequency ($f > 1.5$ Hz) and moderately low tail amplitudes ($0.05 < A_{tail}/L < 0.1$) result in high travelling wave behaviour.

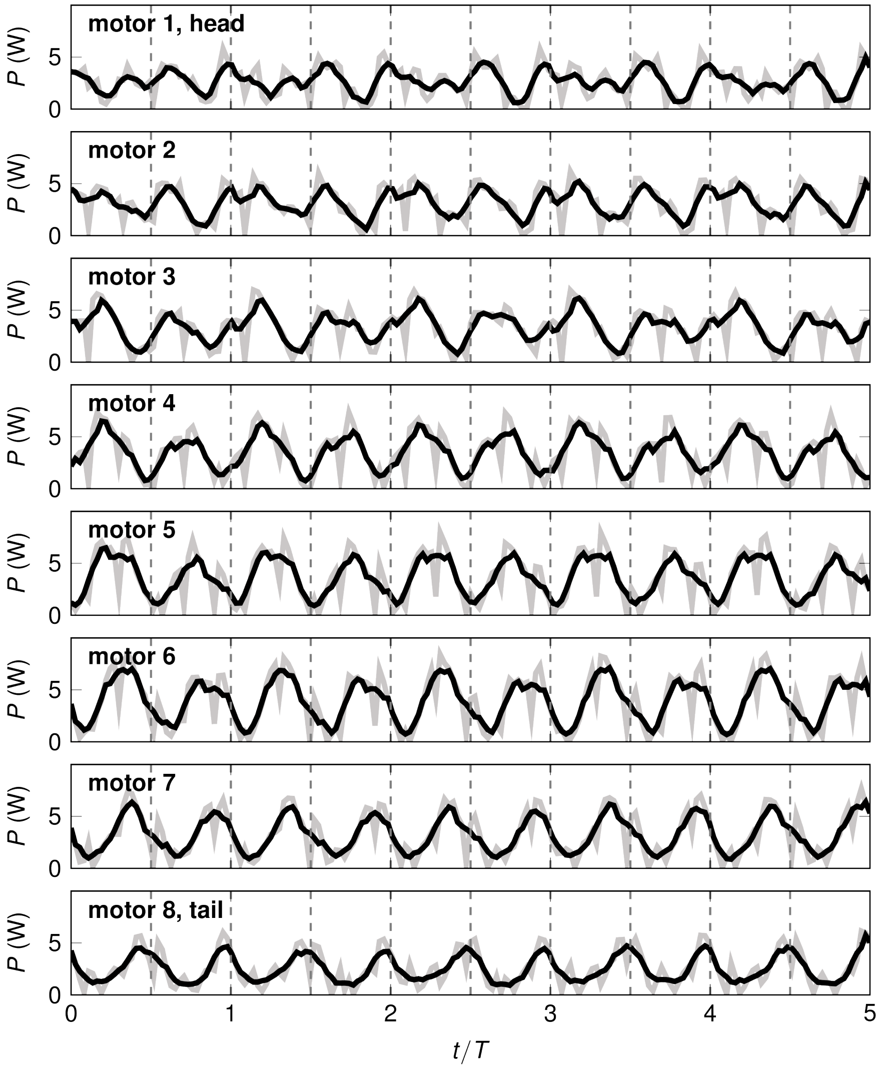

Supplementary Figure S9. Power consumption of robot's motors during motion in water. Raw (grey line) and filtered (black line) power consumption is presented for five periods of motion for all eight motors of the robot. Motors are numbered from 1 to 8, the numbering follows the physical order of the motors along the length of the robot. Motor number one is the motor closer to the head of the robot and motor number eight is the motor closer to the tail of the robot. The power consumption is measured during the kinematics presented and analysed in Fig.1 of the manuscript. The input kinematics have an input wavelength of $\lambda_{\text{input}}/L = 0.71$, joint amplitude $A_{\text{joint}} = 25°$ and a frequency of $f = 1.5\,\text{Hz}$. The motor power consumption varies in time due to the hydrodynamic interactions with the surrounding water during the motion.

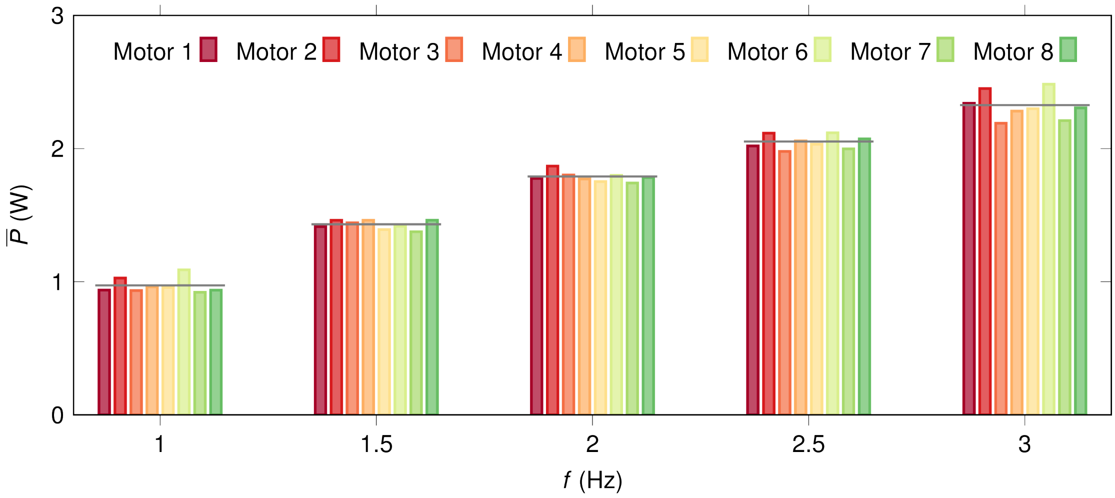

Supplementary Figure S10. Bench test confirms that the individual motors perform similarly. We mechanically decoupled the motors from the shaft and programmed programmed all motors to run the same periodic motion for frequencies ranging from 1 Hz to 3 Hz. The periodic motion consists of a 35° amplitude and the frequency ranges from 1 Hz to 3 Hz. Measurements were obtained for a duration of 30 periods. The average power consumption for the eight motors is presented and coloured according to the motor number. Motor number one is the motor closer to the head of the robot and motor number eight is the motor closer to the tail of the robot. Horizontal lines represent the average power consumption of all eight motors for the frequencies tested. For all frequencies tested the standard deviation of individual motors power consumption remained less than 7.5 % of the average power consumption.
